# Salvage Abdominoperineal Resection for Squamous Cell Anal Cancer: A 30-Year Single-Institution Experience

**DOI:** 10.1245/s10434-018-6483-9

**Published:** 2018-04-24

**Authors:** J. A. W. Hagemans, S. E. Blinde, J. J. Nuyttens, W. G. Morshuis, M. A. M. Mureau, J. Rothbarth, C. Verhoef, J. W. A. Burger

**Affiliations:** 1 Department of Surgical Oncology, Erasmus MC Cancer Institute, University Medical Center Rotterdam, Rotterdam, The Netherlands; 2 Department of Radiation Oncology, Erasmus MC Cancer Institute, University Medical Center Rotterdam, Rotterdam, The Netherlands; 3 Department of Anesthesiology, Erasmus MC, University Medical Center Rotterdam, Rotterdam, The Netherlands; 4 Department of Plastic and Reconstructive Surgery, Erasmus MC Cancer Institute, University Medical Center Rotterdam, Rotterdam, The Netherlands

## Abstract

**Background:**

Failure of chemoradiotherapy (CRT) for anal squamous cell carcinoma (SCC) results in persistent or recurrent anal SCC. Treatment with salvage abdominoperineal resection (APR) can potentially achieve cure. The aims of this study are to analyze oncological and surgical outcomes of our 30-year experience with salvage APR for anal SCC after failed CRT and identify prognostic factors for overall survival (OS).

**Methods:**

All consecutive patients who underwent salvage APR between 1990 and 2016 for histologically confirmed persistent or recurrent anal SCC after failed CRT were retrospectively analyzed.

**Results:**

Forty-seven patients underwent salvage APR for either persistent (*n* = 24) or recurrent SCC (*n* = 23). Median OS was 47 months [95% confidence interval (CI) 10.0–84.0 months] and 5-year survival was 41.6%, which did not differ significantly between persistent or recurrent disease (*p* = 0.551). Increased pathological tumor size (*p* < 0.001) and lymph node involvement (*p* = 0.014) were associated with impaired hazard for OS on multivariable analysis, and irradical resection only (*p* = 0.001) on univariable analysis. Twenty-one patients developed local recurrence after salvage APR, of whom 8 underwent repeat salvage surgery and 13 received palliative treatment. Median OS was 9 months (95% CI 7.2–10.8 months) after repeat salvage surgery and 4 months (95% CI 2.8–5.1 months) following palliative treatment (*p* = 0.055).

**Conclusions:**

Salvage APR for anal SCC after failed CRT resulted in adequate survival, with 5-year survival of 41.6%. Negative prognostic factors for survival were increased tumor size, lymph node involvement, and irradical resection. Patients with recurrent anal SCC after salvage APR had poor prognosis, irrespective of performance of repeat salvage surgery, which never resulted in cure.

Squamous cell carcinoma (SCC) of the anal canal is a relatively rare malignancy, but its incidence has increased over the last few years.^1^ Currently, chemoradiotherapy (CRT) is standard of care for anal cancer, resulting in superior local control compared with radiotherapy alone with 5-year survival rates of 60–80%.^2^–^8^ CRT leads to preservation of the anal sphincter by avoiding surgery. Unfortunately, CRT fails in 20–30% of patients, resulting in persistent (10–15%) or local recurrent disease (10–15%).^2^–^7^,^9^

Salvage abdominoperineal resection (APR) is often the only option for patients with persistent or recurrent anal SCC to achieve durable local control and survival. Several institutes have reported case series on this topic. However, due to heterogeneity in treatment protocols, results on patient outcomes vary widely.^10^–^20^ Our institute has a well-established protocol for treatment of anal SCC, which has changed little in the last three decades. The aims of the present study are to analyze the results of a 30-year experience with salvage APR for recurrent and persistent anal SCC after failed CRT in a large single-center cohort and to identify prognostic factors for overall survival. In addition, outcomes of patients treated for local recurrence developed after primary salvage APR for persistent or recurrent SCC were also analyzed. To the best of the authors’ knowledge, results of repeat surgery for treatment of local recurrence after salvage APR have never been previously studied.

## PATIENTS AND METHODS

Data of all consecutive patients who underwent salvage APR with curative intent for histologically confirmed persistent or recurrent anal SCC between 1990 and 2016 at the Erasmus MC Cancer Institute, a tertiary referral center in The Netherlands, were retrospectively analyzed. Patient demographics, perioperative variables, tumor characteristics, neoadjuvant therapy, short- and long-term outcomes, and postoperative mortality and morbidity were collected from medical records, the municipality register, and general practitioners. All patients were followed up by our institute; last update of follow-up was 22 January 2018. The present study was approved by the Erasmus MC local medical ethics committee (registration number MEC-2017-448).

### Primary Treatment

All primary malignancies were initially treated with radiotherapy, and the majority (78.7%) also received concomitant chemotherapy. Radiotherapy was administered with median dose of 60 Gy [interquartile range (IQR) 60–60 Gy], and chemotherapy was administered in the first four days of the first week [5-fluorouracil (1000 mg/m^2^) and mitomycin C (10 mg/m^2^)]. Patients with histologically proven anal SCC within 6 months after the last day of radiotherapy, or patients with incomplete response, were classified as having persistent disease. Initial complete responders to (chemo)radiotherapy, who were diagnosed with biopsy-proven recurrent anal SCC, after 6 months or more since the last day of radiotherapy, were classified as having recurrent disease.

### Staging

Tumor stage was assessed by physical examination and radiologic imaging according to the American Joint Committee on Cancer (AJCC) tumor–node–metastasis (TNM) staging system (7th edition) for cancer of the anal canal. Nodal stage was assessed by pelvic magnetic resonance imaging (MRI), and suspicious inguinal lymph nodes were biopsied. Computed tomography (CT) scans of the chest and abdomen were used to confirm absence of metastatic disease prior to surgery.

### Surgery

All patients deemed eligible for complete, curative resection underwent salvage APR. Multivisceral resection was performed if necessary. If possible, omentoplasty was performed to fill the pelvis. Primary closure of the perineal defect was routinely performed up to 1999, and if this was not feasible, the open wound was packed for healing by secondary intention. From 2000 onwards, the perineal defect was reconstructed with either a vertical rectus abdominis myocutaneous (VRAM) or gracilis muscle flap.^21^,^22^ Inguinal lymph node dissection was performed in case of biopsy-proven positive lymph nodes. Postoperative complications were graded according to the Dindo–Clavien classification.^23^ Local recurrence after salvage APR was defined as any local recurrence after salvage APR, regardless of whether the indication for salvage APR was for persistent or recurrent anal SCC.

### Statistics

Survival analysis was performed by Kaplan–Meier method, and comparisons were made using log-rank tests. Survival was calculated from day of APR until data of death or last follow-up. Survival rates for recurrence after salvage APR were calculated from date of diagnosis of recurrent anal SCC until death or last follow-up. Cox proportional-hazard models were constructed to identify prognostic factors in univariable and multivariable analysis. Mann–Whitney *U* and chi-squared test were performed as appropriate. Covariables with a trend towards significance (*p* < 0.100) were selected for multivariable analysis, with a maximum of three considering the number of events. Two-sided *p*-values < 0.05 were considered statistically significant. Statistical analysis was performed using IBM SPSS Statistics version 24.0.0 for Windows (IBM Corp, Armonk, New York, USA).

## RESULTS

Forty-seven consecutive patients underwent salvage APR for anal SCC between 1990 and 2016. Patient characteristics are depicted in Table 1.

**Table 1:** Patient and tumor characteristics before and after abdominoperineal resection (*N* = 47)

	*N*	%
Gender		
Male	27	57.4
Female	20	42.6
Age		
At time of diagnosis primary	53 (46–66)*	
At time of operation	56 (48–66)*	
Clinical tumor stage		
T1	8 17.0	
T2	20	42.6
T3	13	27.7
T4	6	12.8
Clinical nodal stage		
N0/Nx	40	85.1
N1	5	10.6
N2	2	4.3
Clinical Metastasis stage		
M0	45	95.7
M+	2	4.3
Histology		
Squamous cell carcinoma	47	100
Pretreatment		
Radiotherapy	47	100
Mean dose Gy	60 (60–60)*	
Concomitant chemotherapy		
5-FU Mitomycin C	36	76.6
5-FU only	1	2.1
No chemotherapy	10	21.3
Indication for surgery		
Persistent disease	24	48.9
Recurrent disease	23	51.1
Time interval radiotherapy and surgery (in months)		
Persistent disease	5 (4–7)*	
Recurrent disease	15.0 (9.5–37.5)*	
Surgical procedure		
APR	35	74.5
APR and posterior vaginal wall	4	8.5
Posterior exenteration	4	8.5
Total pelvic exenteration	2	4.3
Posterior exenteration and vulvectomie	2	44.3
Additional procedures		
Partial sacrectomy	2	4.3
Synchronous ILND	2	4.3
Omentoplasty	33	70.2
IORT	2	4.3
Wound closure and/or reconstruction		
Primary closure	10	21.3
Wound left open	1	2.1
VRAM-flap	31	66.0
Gracilis flap	3	6.4
Pudendus flap	1	2.1
Gluteal flap	1	2.1
Operating time		
Minutes	378.6 ± 129.9**	
Pathological tumor size		
Maximum diameter (millimeter)	30.0 (20.0–48.3)*	
Pathological nodal stage		
N0/Nx	41	87.2
N1	2	4.3
N2	4	8.5
Pathological metastases stage		
M0/Mx	43	91.5
M1	4	8.5
Vasoinvasion		
Yes	11	23.3
No	18	38.3
Unknown	18	38.3
Perineural growth		
Yes	14	29.8
No	15	31.3
Unknown	18	38.3
Pathological resection margins		
R0	38	80.9
R1	8	17.0
R2	1	2.1

*Median and interquartile range, **Mean and standard deviation

*APR* abdominoperineal resection, *IORT* intra-operative radiotherapy, *VRAM* vertical rectus abdominus muscle, *ILND* Inguinal lymph node dissection, *5-FU* 5-fluorouracil

### Surgical Results

Indications for surgery were either persistent (*n* = 24; 48.9%) or recurrent disease (*n* = 23; 51.1%). Median time between the last day of (chemo)radiotherapy and date of surgery was 5 months (IQR 4–7 months) for patients with persistent disease and 15 months (IQR 9.5–37.5 months) for patients with recurrent disease. APR without additional resections was performed in 35 patients, APR with posterior vaginal wall resection in 4 patients, posterior exenteration in 6 patients (including vulvectomy in 2 patients), and total pelvic exenteration in 2 patients. Other additional procedures were partial sacrectomy (*n* = 2), synchronous inguinal lymph node dissection (*n* = 2), and intraoperative radiotherapy (IORT, *n* = 2). Omentoplasty was performed in 33 patients. One patient had two lesions in the liver suspicious for metastases, which were histopathologically confirmed by frozen section. Salvage APR was performed, but the liver metastases were not resected. Until 1999, primary perineal closure was performed in seven patients, one open wound was packed for secondary healing, and one gluteal transposition flap was performed for reconstruction. In 38 patients treated from 2000 onwards, primary perineal closure was performed three times, while a locoregional flap for perineal closure was used 35 times [VRAM flap (*n* = 31), gracilis muscle flap (*n* = 3), and bilateral pudendal flap (*n* = 1)]. Surgical characteristics are presented in Table 1. Radical resection (R0) was achieved in 38 patients (80.9%), microscopically irradical resection (R1) in 8 patients (17.0%), and macroscopically irradical resection (R2) in 1 patient (2.1%). One patient had liver metastases, and three patients had inguinal lymph node metastases. Tumor characteristics are listed in Table 1.

### Mortality and Morbidity

None of the patients died within 30 days of surgery. Within 2 months, there was one case of euthanasia due to unbearable suffering from severe wound infection and no perspective of cure considering confirmed liver metastases. The majority of patients (*n* = 33; 70.3%) experienced no or minor complications (Dindo–Clavien ≤ 2), and 14 patients (29.7%) developed major complications (Dindo–Clavien ≥ 3). Mortality and morbidity are displayed in Table 2. Six out of 10 patients with primary closure of the perineal defect and 9 out of 36 patients with muscle flap reconstruction (MFR) experienced perineal wound complications. Nine patients required surgery for perineal wound complications. The latter were treated with debridement with (*n* = 5) or without vacuum-assisted closure therapy (*n* = 2) and muscle flap necrosectomy followed by repeat reconstruction (*n* = 2). Median time between last day of radiotherapy and surgery did not significantly influence perineal wound complications (*p* = 0.909). The proportion of patients with perineal wound complications was lower in patients treated with MFR (25%; 9/36) compared with patients treated without MFR (54.5%; 6/11), however this was not significant (*p* = 0.066).

**Table 2:** Mortality, morbidity, and perineal wound complications

	*N*	%
Mortality		
< 30 days after surgery	0	0
During hospital admission	1	2.1
Dindo-Clavien		
None	17	36.2
Dindo 1	6	12.8
Dindo 2	10	21.3
Dindo 3A	1	2.1
Dindo 3B	10	21.3
Dindo 4	3	6.4
Dindo 5	0	0
Major complications		
Pulmonary embolism	1	2.1
Aspiration pneumonia	2	4
Gastric ulcer bleeding	1	2.1
Major complications requiring surgery		
Stoma necrosis	1	2.1
Abdominal wound necrosis	1	2.1
Fascia dehiscence	1	2.1

Perineal wound complications	MFR (*N* = 36)	No MFR (*N* = 11)
Additional muscle flap reconstruction	1	1
Vacuum assisted therapy	3	2
Wound complication treated conservative	4	3
Wound complication requiring debridement	2	0
Perineal hernia	1	1

*MFR* muscle flap reconstruction

### Survival

**Fig. 1:**
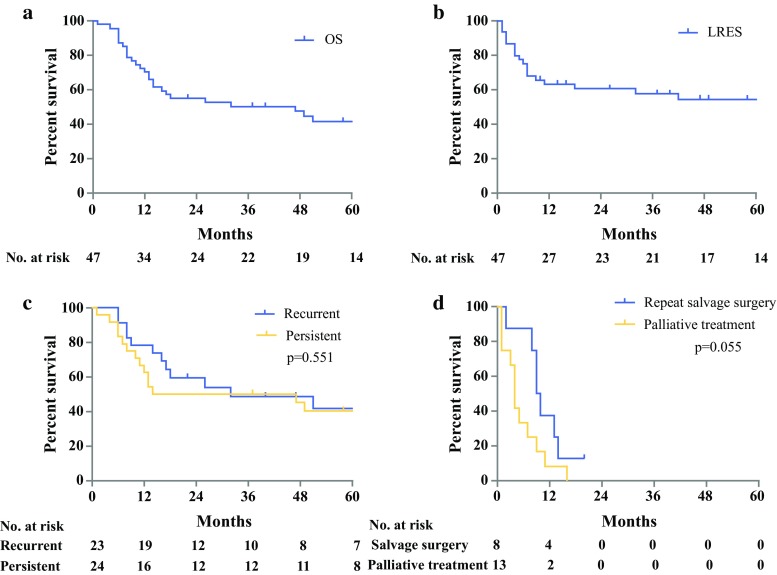
**a** Overall survival (OS). **b** Local recurrence-free survival (LRFS). **c** OS for persistent versus recurrent disease. **d** OS for local recurrence after salvage APR; repeat salvage surgery versus palliative treatment

**Fig. 2:**
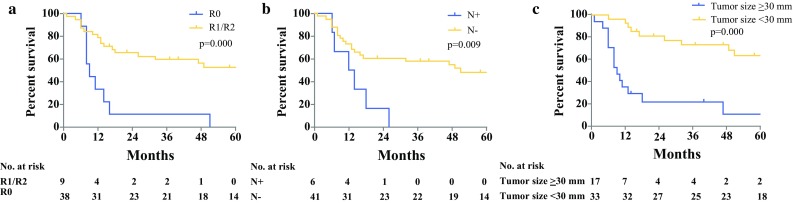
Overall survival curves (prognostic factors): **a** resection margin, **b** nodal stage, and **c** pathological tumor size (diameter in millimeters with median as cutoff value)

Median follow-up time was 80 months (95% CI 68.6–91.4 months). At last follow-up, 19 patients (40.4%) were alive. Median overall survival (OS) was 47 months (95% CI 10.0–84.0 months), and the estimated 5-year survival rate was 41.6%. Survival curves did not differ significantly between patients with persistent versus recurrent disease (5-year survival rate 40.4 vs. 41.7%, respectively; *p* = 0.551). Survival curves are shown in Fig. 1. On both univariable and multivariable analysis, increased pathological tumor size (*p* < 0.001) and positive lymph nodes (*p* = 0.014) were significantly associated with worse OS. Irradical resection was only significantly associated on univariable analysis (*p* = 0.001) but not on multivariable analysis (*p* = 0.087). Analyses are presented in Table 3, and the influence on survival in Fig. 2.

**Table 3:** Univariable and multivariable survival analysis for overall survival of squamous cell carcinoma

	Univariable	*P* value	Multivariable	*P* value
	Hazard ratio [95% CI]		Hazard ratio [95% CI]	
Male versus female	1.150 [0.536–2.466]	0.720	–	–
Age at time of operation	1.021 [0.986–1.058]	0.239	–	–
CTxRTx versus RTx	0.884 [0.332–2.351]	0.805	–	–
Recurrent disease versus persistent disease	0.794 [0.794–1.709]	0.556	–	–
Multivisceral resection	1.169 [0.524–2.608]	0.704	–	–
Irradical resection (R1/R2)	4.056 [1.746–9.423]	0.001	2.786 [0.862–9.005]	0.087
Node positive (N1/N2)	3.228 [1.255–8.302]	0.015	4.445 [1.356–14.563]	0.014
Metastasis positive (M1)	2.603 [0.878–7.712]	0.084	–	–
Vasoinvasion	2.081 [0.795–5.679]	0.144	–	–
Perineural growth	2.702 [0.973–7.504]	0.056	–	–
Pathological tumor size (maximum diameter in mm)	1.039 [1.023–1.055]	< 0.001	1.036 [1.018–1.054]	< 0.001

*CTxRTx* chemoradiotherapy, *RTx* radiotherapy

### Recurrence after Salvage APR

The overall rate of disease recurrence after salvage APR was 55.3%. Twenty-one patients (44.7%) developed local recurrence after salvage APR, including 13 patients with simultaneous locoregional recurrence or distant metastases [inguinal lymph node (*n* = 7), liver (*n* = 2), adrenal gland (*n* = 1), retroperitoneal lymph nodes (*n* = 1), peritoneal carcinomatosis (*n* = 1), and cervical lymph node + liver metastasis (*n* = 1)]. Five patients developed distant metastases or locoregional recurrence only [inguinal lymph node (*n* = 2), retroperitoneal lymph nodes (*n* = 1), hilar lymph nodes (*n* = 1), liver metastases (*n* = 1)]. Median OS for patients with local recurrence and/or distant metastases after salvage APR was 12 months (95% CI 8.3–15.7 months). Median local-recurrence-free survival after salvage APR (LRFS) was not reached. The estimated 5-year LRFS after salvage APR was 51.1%. None of the patients developed local recurrence after 42 months from salvage APR. Three patients received postoperative chemotherapy for metastatic disease, and none of the patients received standard adjuvant chemotherapy.

Eight patients with local recurrence after salvage APR underwent repeat salvage surgery by extensive local excision, including additional inguinal lymph node dissection (*n* = 2), liver metastases resection (*n* = 1), and cervical lymph node dissection (*n* = 1).

Thirteen patients underwent palliative treatment for local recurrence after salvage APR, including fistula resection (*n* = 2), radiotherapy in combination with hyperthermia (*n* = 2), and chemotherapy for metastatic disease (*n* = 2), while seven patients received best supportive care only. Median OS for all patients with local recurrence after salvage APR, calculated from date of diagnosis of local recurrence, was 7 months (95% CI 1.0–13.0 months). The 1-year survival rate was 19.0%, and all patients died within 15 months except for one patient, who had undergone repeat salvage surgery and was still alive at last follow-up of 22 months.

There was no significant difference (*p* = 0.055) in survival of patients with local recurrence after salvage APR treated with repeat salvage surgery, with median OS of 9 months (95% CI 7.2–10.8 months), compared with patients with palliative treatment, with median OS of 4 months (95% CI 2.8–5.1 months).

## DISCUSSION

The present study describes the results of salvage APR for SCC of the anal canal after failure of initial primary therapy in 47 patients. Overall estimated 5-year survival was 41.6%. Negative prognostic factors were increased pathological tumor size and lymph node involvement on multivariable analysis, and positive resection margin only on univariable analysis. Type of local failure did not affect survival. The overall local recurrence rate after salvage APR was 44.7%. None of the patients who developed local recurrence after salvage APR could be cured, and all had poor prognosis.

Although surgery has been replaced by CRT for primary treatment of SCC of the anal canal, salvage APR has remained the gold standard for patients with persistent disease or local recurrent disease after failed CRT. Due to the relative rarity of the procedure for this indication, most published series consist of only a small number of patients treated over a long period of time, and are therefore prone to a certain degree of bias. We present herein a rather homogeneous group of patients. All patients were treated with an adequate radiation dose of > 45 Gy, and all but eight patients received the standard protocol of 60 Gy. This in contrast to some other published series where the study population was treated with a wide range of radiation doses.^9^–^11^,^16^

The percentages of radical resection and 30-day postoperative mortality are comparable to previous studies.^9^,^13^,^24^–^27^ Outcome measures of complications after salvage APR varied widely in other studies, preventing adequate comparison. However, in the current study, surgical reinterventions were slightly more common (25.5%) than the range reported by others (12–20%).^13^,^24^–^26^ In this study, 31.9% of patients experienced perineal complications, while others reported perineal complications in 22–50% of patients, regardless of use of muscle flap reconstruction.^9^,^13^,^16^,^25^,^27^ We could not identify a group prone to perineal complications based on time between radiotherapy and surgery or use of muscle flap reconstruction, possibly due to small numbers.

The 5-year OS in this study of 41.6% lies within the range of 23–69% reported by other authors. Survival of patients with persistent disease did not differ significantly from that of patients with recurrent disease, which is also in agreement with results published previously,^10^,^11^,^14^,^15^,^17^,^19^,^28^ although some studies did report poorer survival rates in patients with persistent compared with recurrent disease.^10^,^16^ This could be explained by more aggressive behavior of tumor cells in persistent disease or fast regrowth. However, other studies reported significantly worse survival in patients with recurrent disease, which could not be explained clearly.^29^,^30^

We found that increased pathological tumor size, lymph node involvement, and positive resection margins adversely affected survival, which is in concordance with most other series (Appendix 1).^9^–^11^,^13^–^15^,^17^,^24^,^25^,^28^,^30^–^32^ Although not identified on multivariable analysis in the present study, positive resection margin seems to remain the most common factor negatively affecting survival. These findings emphasize the importance of achieving negative resection margins, which can sometimes only be achieved by aggressive multivisceral resection or multidisciplinary treatment.

Recently, Hallemeier et al.^30^ reported a multidisciplinary approach, including reirradiation with or without concomitant chemotherapy and IORT, in a small group of patients with persistent or recurrent anal cancer. Only 21% developed recurrence within the reirradiated area. The 5-year OS was 23%, but they specifically treated patients with expected narrow or positive resection margins.^30^ In the present study, only two patients received IORT, and none received reirradiation prior to salvage surgery, because of the high-dose radiotherapy used as primary treatment. Wright et al.^33^ retrospectively analyzed 14 patients with locoregional recurrent anal SCC who underwent salvage surgery and IORT. Addition of IORT was not associated with locoregional control or survival benefit and did not compensate for positive surgical margins.^33^ Reirradiation and IORT could potentially decrease local recurrence rate, but this remains unclear.

Currently there is no role for standard adjuvant chemotherapy, however the combination of cisplatin and 5-fluorouracil (5-FU) is the gold standard in metastatic disease, with an overall response rate of 60%.^34^,^35^ Eng et al.^36^ showed a prolonged OS for multidisciplinary management with systemic chemotherapy and intervention compared with palliative chemotherapy only in patients with unresectable and metastatic anal SCC.^36^ In the present study, three patients received postoperative chemotherapy without additional intervention. Therefore, we could not clearly assess the effect on OS. Multidisciplinary treatment for unresectable and metastatic anal SCC can potentially lead to prolonged OS.

To our knowledge, this is the first study to present data on treatment of recurrent anal SCC after failed CRT for primary anal SCC and salvage APR for recurrent/persistent anal SCC. Alamri et al.^27^ and Correa et al.^24^ only reported survival for these patients. Some patients with local recurrence after salvage APR also had distant metastases or locoregional recurrence, and type of surgery was not protocolled as it is for the primary salvage APR. On the other hand, our results clearly show that recurrence after salvage APR has poor prognosis, regardless of the treatment. Palliative surgery may still be considered for some patients, especially those with pain. Cure, however, does not seem to be possible.

This study is limited by its retrospective nature and the small number of patients collected over a long time period. Patients with persistent or recurrent disease have different tumor biology, and mixing these cases could affect the outcomes of salvage APR. Advances in diagnostic imaging and treatment were made during the study period and likely contributed to heterogeneity in our study population and outcomes.

## CONCLUSIONS

The results of the present study show that salvage APR for patients with SCC of the anal canal after failed CRT provides adequate long-term survival and local control. Prognostic factors for survival were advanced tumor stage, lymph node involvement, and positive resection margins. Patients with recurrent anal SCC after salvage APR had poor prognosis irrespective of performance of repeat salvage surgery, which never resulted in cure.

## Appendix 1: Overview of Current Literature

**Table:**

Ref.	Year of publication	No. of patients	5-Year OS (%)	Prognostic factors for OS after salvage APR
Zelnick et al.^20^	1992	9	24	Not identified or not mentioned
Ellenhorn et al.^11^	1993	38	44	Nodal disease
				Tumor fixed to lateral pelvic wall
				Involvement of perirectal fat
Longo et al.^6^	1994	34	23–53	Stage
				Method of treatment
Pocard et al.^37^	1998	21	33	Not identified or not mentioned
Allal et al.^29^	1999	26	45	Not identified or not mentioned
Smith et al.^18^	2001	22	33	Not identified or not mentioned
Van der Wal et al.^19^	2001	17	47	Not identified or not mentioned
Nilsson et al.^16^	2002	35	52	Persistent disease
Akbari et al.^10^	2004	62	33	Tumor size > 5 cm
				Local extent
				Nodal disease
				Positive resection margins
Ghouti et al.^12^	2005	36	69	Not identified or not mentioned
Ferenschild et al.^38^	2005	18	30	Not identified or not mentioned
Renehan et al.^9^	2005	73	40	Positive resection margins
Mullen et al.^15^	2006	31	64	Nodal disease
				< 55 Gy radiotherapy dose
Stewart et al.^31^	2007	22	24–48	Tumor differentiation
				Positive resection margins
Schiller et al.^17^	2007	40	39	Tumor size
				Sex (male)
Mariani et al.^14^	2008	83	57	Age > 55 years
				Nodal disease
				T3–4 tumor
				Local extent
Sunesen et al.^25^	2009	49	61	Positive resection margins
Eeson et al.^32^	2011	51	29	Positive resection margins
Correa et al.^24^	2012	111	25	Nodal disease
				Positive resection margin
				Perineural and/or lymphovascular invasion
Lefevre et al.^13^	2012	105	61	T3–T4 status
				Positive resection margins
				Metastatic disease
Hallemeier et al.^30^	2014	32	23	Recurrent disease versus persistent disease
				Positive resection margins
				Viable disease in resection specimen
Alamri et al.^27^	2016	27	78	None identified
Pesi et al.^26^	2017	20	37	None published
Present study	2017	47	41	Increased pathological tumor size (mm)
			–	Nodal disease
			–	Positive resection margins
